# Platform-Specific Fc *N*-Glycan Profiles of an Antisperm Antibody

**DOI:** 10.3390/antib13010017

**Published:** 2024-03-06

**Authors:** Ellena Nador, Chaoshuang Xia, Philip J. Santangelo, Kevin J. Whaley, Catherine E. Costello, Deborah J. Anderson

**Affiliations:** 1 Department of Medicine, Boston University Chobanian & Avedisian School of Medicine, Boston, MA 02118, USA; 2 Center for Biomedical Mass Spectrometry, Boston University Chobanian & Avedisian School of Medicine, Boston, MA 02118, USA; 3 Wallace H. Coulter Department of Biomedical Engineering, Emory University, Atlanta, GA 30322, USA; 4 ZabBio, Inc., San Diego, CA 92121, USA

**Keywords:** monoclonal antibodies, *N*-glycosylation, Fc receptor, contraception, manufacturing, mRNA, mass spectrometry

## Abstract

IgG Fc *N*-glycosylation is necessary for effector functions and is an important component of quality control. The choice of antibody manufacturing platform has the potential to significantly influence the Fc glycans of an antibody and consequently alter their activity and clinical profile. The Human Contraception Antibody (HCA) is an IgG1 antisperm monoclonal antibody (mAb) currently in clinical development as a novel, non-hormonal contraceptive. Part of its development is selecting a suitable expression platform to manufacture HCA for use in the female reproductive tract. Here, we compared the Fc glycosylation of HCA produced in two novel mAb manufacturing platforms, namely transgenic tobacco plants (*Nicotiana benthamiana*; HCA-N) and mRNA-mediated expression in human vaginal cells (HCA_mRNA_). The Fc *N*-glycan profiles of the two HCA products were determined using mass spectrometry. Major differences in site occupancy, glycan types, and glycoform distributions were revealed. To address how these differences affect Fc function, antibody-dependent cellular phagocytosis (ADCP) assays were performed. The level of sperm phagocytosis was significantly lower in the presence of HCA-N than HCA_mRNA_. This study provides evidence that the two HCA manufacturing platforms produce functionally distinct HCAs; this information could be useful for the selection of an optimal platform for HCA clinical development and for mAbs in general.

## 1. Introduction

Glycosylation is the most common post-translational modification (PTM) of monoclonal antibodies (mAbs) and has a significant role in their biological activity, stability, and antigenicity [1,2,3]. Glycosylation can alter the pharmacokinetics and pharmacodynamics of an antibody through interactions with the neonatal Fc receptor, the endocytic mannose receptor, the asialoglycoprotein receptor, and other receptors [4]. Glycans on mAbs expressed by platforms such as non-human mammalian cell lines have proven to cause unwanted immunogenic responses [5]. Thus, glycan analysis is crucial to the quality control process of therapeutic antibodies, though there is little clarity over the extent to which glycosylation in this context should be regulated [6].

**Figure 1:**
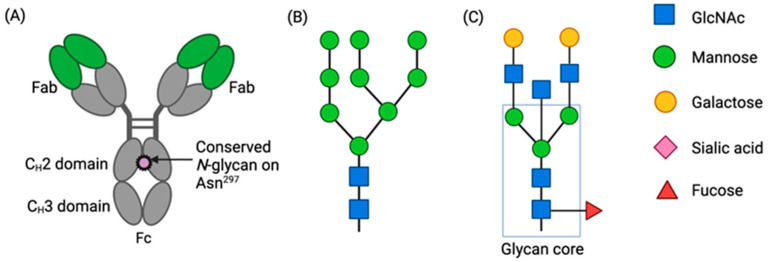
The conserved IgG Fc *N*-glycan. (**A**) Structure of an IgG antibody. (**B**) *N*-glycan precursor. (**C**) Example of a mature biantennary *N*-glycan with a bisecting GlcNAc. The rectangle indicates the trimannosyl chitobiose glycan core. According to the international agreed convention [10], symbols used in this figure and in Figure 4 to represent glycan moieties are shown to the right: hexose residues are circles, *N*-acetyl hexose residues are squares, deoxy hexose residues are triangles, and the purple diamond is *N*-acetylneuraminic acid. Created with Biorender.com.

The majority of monoclonal antibodies available on the market are of the isotype subclass IgG1 [7]. Human IgGs contain a conserved *N*-linked glycosylation site at asparagine residue 297 (Asn^297^) in the heavy chain constant domain 2 (CH2) of the Fc region as a crucial element in IgG structure and function [8] (Figure 1A). *N*-linked glycosylation features a distribution of oligosaccharides consisting of a varying number of sugar moieties attached to the amide nitrogen of an asparagine residue via a trimannosyl chitobiose core. In humans, these sugar moieties are limited to fucose, mannose, *N*-acetylglucosamine (GlcNAc), galactose, and sialic acid residues. The *N*-glycan lipid precursor, dolichol phosphate, is synthesized in the endoplasmic reticulum (ER) [9]. Subsequent transferase reactions complete the assembly of the *N*-glycan precursor Glc_3_Man_9_GlcNAc_2_ on the dolichol phosphate. The precursor glycan is transferred to nascent proteins at an asparagine residue within the sequence Asn-X-Ser/Thr, where X is any amino acid except proline. The glucose residues are removed by the sequential actions of ER glucosidases to leave a Man9 structure (Figure 1B). Proteins are subject to proper folding and may undergo some trimming of mannose residues within the ER before transport to the Golgi for *N*-glycan maturation. Here, the stepwise actions of highly selective glycosidases and glycotransferases can replace mannose branches with GlcNAc, and additional glycan moieties that may include fucose, galactose, and sialic acid can be added to generate glycan structures such as that shown in Figure 1C. The mature *N*-glycan falls into one of the following three main classes: oligomannose, complex, or hybrid [10].

Several antibody effector functions are facilitated by the Fc region, including opsonization, antibody-dependent cellular phagocytosis (ADCP), and complement-dependent cytotoxicity (CDC). Depending on the therapeutic goal of an antibody, these effector functions may be desirable or undesirable. While effector functions are beneficial for the efficient clearing of antigens, an exceedingly strong and persistent immune response, especially in sensitive mucosal areas like the vagina, can be damaging. *N*-glycosylation is a key factor that modulates IgG Fc-mediated functions. The IgG-Fc glycan at residue 297 is necessary for an effector function through the Fcγ receptor (FcγR) binding [8]. Since the Fc region is the site of contact for Fc receptors, changes in the structure of the Fc domain conferred by the conserved Fc *N*-glycan at Asn^297^ can have a major impact on receptor binding [11]. Moreover, certain glycan moieties can affect binding to FcγRs differently and thus influence an antibody’s potential for Fc-mediated functions [12,13,14]. Overall, Fc *N*-glycans can play a significant role in fine-tuning the pharmaceutical properties of an antibody. Efforts in glycoengineering are of increasing interest as a means to customize therapeutic antibody effector functions [15].

The main contraceptive mechanism of the Human Contraception Antibody (HCA), an IgG1 antisperm mAb, in development as a novel non-hormonal contraceptive, is sperm agglutination. HCA binds the male reproductive tract-specific glycoprotein CD52g. However, one Fc-mediated function of HCA that may have important implications is ADCP. We previously demonstrated that HCA is capable of mediating sperm phagocytosis via Fc-FcγR interactions [16]. Though ADCP could potentially serve as a secondary contraceptive mechanism to clear sperm, subsequent sperm antigen presentation, which may be possible due to the presence of antigen-presenting cells within the vaginal epithelium [17,18], is undesirable. Various Fcγ receptors are responsible for eliciting ADCP, including FcγRI, FcγRlla, and FcγRIII [19]. Interactions between platform-specific HCA Fc *N*-glycans and these Fcγ receptors may modulate HCA-mediated sperm phagocytosis and antigen presentation, potentially affecting the clinical profile of the antibody.

**Figure 2:**
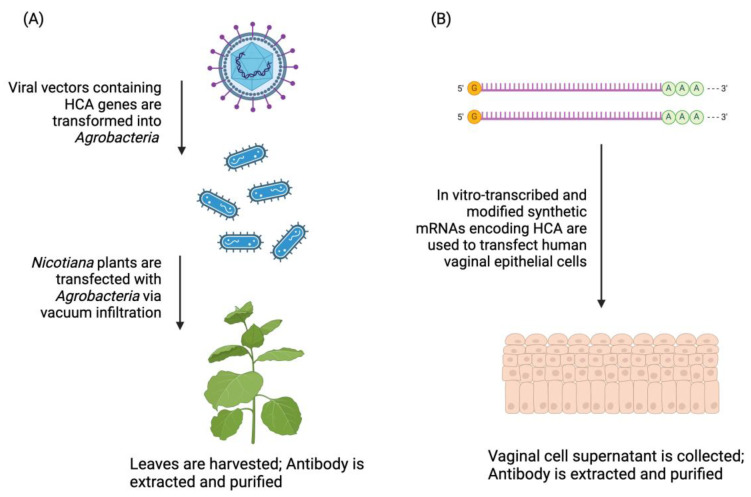
Analysis of products from novel HCA expression platforms. (**A**) HCA produced in *Nicotiana benthamiana* (HCA-N). (**B**) HCA produced in mRNA-transfected vaginal epithelial cells (HCA_mRNA_). Created in Biorender.com.

Advances in monoclonal antibody engineering have led to the exploration of novel production methods to increase yield and lower costs. Our group has explored several expression systems for HCA. One such platform is *Nicotiana benthamiana*, a close relative of the tobacco plant, for the production of “plantibodies” [20,21]. This platform allows for the rapid, low-cost, large-scale production of monoclonal antibodies. It is a viral-based, transient expression system that leads to the accumulation of antibodies within days (Figure 2A). Whole mature plants (genetically modified to knock out xylosyl- and α1,3-fucosyl-transferases) are infiltrated with a highly diluted *Agrobacterium* suspension carrying t-DNA encoding viral replicons. The result is a high copy number of RNA molecules encoding the antibody. Because the *Nicotiana* plants used in this system are transgenic strains with altered glycosylation pathways, the expressed antibodies contain mammalian glycoforms. This production method is currently being used to manufacture HCA-formulated topical vaginal films for clinical trials [22,23].

Another platform for HCA is synthetic mRNA encoding HCA in vivo in the female reproductive tract (FRT) (Figure 2B). mRNA platforms provide several advantages, including cost-effectiveness, the scalability of mRNA production, high efficiency, reversibility, safety, and durability [24]. Synthetic mRNAs encoding HCA are generated via in vitro transcription and modified with a 5′ cap and N1-methylpseudouridine substitution to increase stability and evade innate immune sensors [25]. The HCA mRNA strands are then used to transfect vaginal epithelium; once mRNA is taken up by the cells, host ribosomal machinery translates and secretes HCA. This method was previously established for the in vivo expression of anti-RSV antibodies in the lung [26] and anti-HIV antibodies in vaginal mucosa [27]. mRNA HCA expression within the vaginal tract bypasses the need to eliminate non-human glycans, as is required in *Nicotiana* expression. mRNA uptake in human vaginal cells leads to antibody production with glycans native to the host.

Glycosylation is one of the most critical quality attributes that impact the efficacy, safety, and stability of monoclonal antibody therapeutics. It is cell-type-dependent, inherently heterogeneous, and relies on a number of factors that contribute to the final structure, such as enzyme levels within a cell, the availability of monosaccharide nucleotides, and Golgi architecture [28]. Thus, the choice of production platform and consequent types of PTMs can dramatically impact the biophysical properties of antibodies in the solution. Antibody production in different systems can result in a variety of heterogeneous glycoforms at site Asn^297^. For example, the most widely used platform for biopharmaceutical production, the Chinese hamster ovary cell (CHO), yields heterogeneous glycans, which can lead to inconsistent function [29]. With novel expression systems emerging, the extent to which their respective glycosylation patterns affect mAb function is important to understand.

We applied mass spectrometry-based glycoproteomic techniques to characterize and compare the HCA Fc *N*-glycan compositions on HCA produced by two current expression systems, namely *Nicotiana benthamiana* and synthetic mRNA in human vaginal cells. We demonstrated that differences in the Fc *N*-glycan site occupancy and glycoform distributions influenced the bioactivity of HCA, specifically antibody-dependent cellular phagocytosis, an Fc-mediated function. 

## 2. Materials and Methods

### 2.1. mRNA HCA Production (HCA_mRNA_VK2_)

VK2 E6/E7 (human vaginal epithelial) cells [30] were obtained from ATCC and cultured in a complete Keratinocyte-Serum Free medium supplemented with the human recombinant epidermal growth factor (rEGF), bovine pituitary extract (BPE), and Pen-Strep (GIBCO-BRL 17005-042). Synthetic mRNA was generated via in vitro transcription and used to transfect cells as previously described [27]. Briefly, mRNA strands encoding heavy and light chains of HCA were generated by ordering sequences as DNA gBLocks with 5′ and 3′ UTRs, which were cloned into a vector. Vectors were purified and in vitro transcribed (IVT) with an N1-methyl-pseudouridine modification. Resultant mRNAs were purified and capped prior to use. The Lipofectamine MessengerMAX reagent (Thermo Fisher Scientific, Inc., Waltham, MA, USA) was used to transfect VK2 cells with the indicated amount of mRNA in Opti-MEM (Gibco) per T75 flask. Following a two-day incubation at 37 °C, IgG molecules (HCA_mRNA_VK2_, shorthand HCA_mRNA_) were purified from the cell supernatant using tangential flow filtration (TFF) and a 100K molecular weight cutoff cartridge for functional analysis. 

### 2.2. Nicotiana HCA Production (HCA-N)

HCA was produced in *Nicotiana benthamiana* (HCA-N) by KBio, Inc. (Owensboro, KY, USA) as previously described [31,32]. Transgenic strains of *Nicotiana* plants subjected to fucosyl- and xylosyl-transferase knockout (ΔXF) were used [33]. Xylosyl-transferase (XylT) knockout prevents the addition of xylose, a non-mammalian glycan residue. The knockout of α1,3-fucosyltransferase (FucT) prevents core α1,3-fucose, which is a non-mammalian linkage of fucose. Briefly, whole mature plants were vacuum-infiltrated with an *Agrobacteria* suspension carrying t-DNAs encoding viral replicons, resulting in a high copy number of RNA molecules encoding HCA. The plants were then harvested to extract and purify HCA-N. 

### 2.3. Mass Spectrometry Analysis

The HCA IgG protein was characterized by nano ultra-high-performance liquid chromatography-tandem mass spectrometry (nanoUPLC-MS and MS/MS). Prior to analysis, each IgG sample (5 µg) was reduced with 10 mM of dithiolthreitol, alkylated with 50 mM of iodoacetamide, and then digested with Glu-C (C-terminal cleavage of glutamic acid, 1:50, enzyme:protein) and trypsin (C-terminal cleavage at arginine and lysine, 1:50, enzyme:protein) at 37 °C for 16 h and the digest mixture was cleaned up with a C18 SPE cartridge. Next, samples were analyzed by nanoUPLC-MS/MS for the determination of peptide molecular masses followed by fragmentation in order to locate the glycosylation site(s) and assign glycoform compositions. For the determination of the *N*-glycosylation site occupancy, an aliquot of the above-digested mixture was dissolved in water (^18^O, 97%, Cambridge Isotope Laboratories, Inc., Tewksbury, MA, USA) containing 50 mM of NH_4_HCO_3_ and deglycosylated with PNGase F (New England Biolabs Inc., Ipswich, MA, USA) at 37 °C for 16 h prior to MS analysis. NanoUPLC-MS/MS analyses were performed on an Orbitrap Fusion Lumos Tribrid mass spectrometer (Thermo Fisher Scientific, Waltham, MA, USA) coupled with an ACQUITY UPLC M-Class system (Waters Corp., Milford, MA, USA) via a TriVersa NanoMate (Advion, Ithaca, NY, USA). A nanoEase Symmetry C18 UPLC Trap Column (100 Å, 5 μm, 180 μm × 20 mm, Waters) was used as the trapping column, and a nanoEase MZ HSS C18 T3 UPLC column (100 Å, 1.8 μm, 75 μm × 100 mm, Waters) was used as the analytical column for LC separation. Peptides were trapped at 4 μL/min for 4 min with 1% acetonitrile and 0.1% formic acid (Solvent A). Then, the peptide/glycopeptide mixtures were separated on an analytical column using different concentrations of 99% acetonitrile/0.1% formic acid (Solvent B): 0–1 min: 2% B, 1–3 min: 2–5% B, 3–43 min: 5–40%.

MS analyses were performed in the positive mode with the radio frequency (RF) lens set to 30%, and scans were acquired with the following settings: a 120,000 resolution @ *m*/*z* 200, a scan range of *m*/*z* 370–2000, 1 μscan/MS, a normalized AGC target at 250%, and a maximum injection time of 50 ms. For high energy collisional dissociation (HCD) analyses, initial MS^2^ scans (normalized collision energy (NCE) 30%) were acquired with the following settings: a 15,000 resolution @ *m*/*z* 200, a scan range of *m*/*z* 100–2000, 1 μscan/MS, AGC target 1 × 10^6^, and a maximum injection time of 100 ms. For the analysis of samples that had not been treated with PNGase F, oxonium ions were used to sense the presence of a glycopeptide and then trigger the generation of an HCD MS/MS spectrum. If two of six common oxonium ions (*m*/*z* 204.0867 (HexNAc ion), *m*/*z* 138.0545 (HexNAc-CH_6_O_3_ ion), *m*/*z* 366.1396 (HexNAcHex ion), *m*/*z* 168.0653 (HexNAc—2 H_2_O fragment ion), *m*/*z* 186.0760 (HexNAc—H_2_O fragment ion), *m*/*z* 292.1031 (NeuAc ion), *m*/*z* 274.0927 (NeuAc—H_2_O fragment ion) were detected in the HCD spectrum within 15 ppm of mass tolerance, MS^2^ spectra were acquired. For HCD-triggered HCD, the triggered-HCD scan was set to a 30,000 resolution @ *m*/*z* 200, a scan range of *m*/*z* 100–2000, 1 μscan/MS, AGC target 1 × 10^6^, and a maximum injection time of 150 ms. MS/MS data were searched against 20,352 entries in a UniProtKB database restricted to *Homo sapiens* (downloaded in May 2021) by PMI-Byonic (version v3.8-11, Protein Metrics Inc., Cupertino, CA, USA). Carbamidomethylation (Cys) was set as a fixed modification, whereas Met oxidation and protein N-terminal acetylation were defined as variable modifications. Mass tolerance was set to 10 and 20 ppm at the MS and MS/MS levels, respectively. Enzyme specificity was set to the C-terminal of glutamic acid and C-terminal of arginine and lysine, with a maximum of two missed cleavages. The Protein Metrics 132 human *N*-glycan library was used for the assignment of *N*-linked glycosylation. All assignments were verified by manual inspection. For samples that had been treated with PNGase F, HCD MS/MS data were obtained for the 20 most abundant peaks in each MS1 spectrum. Ratios of unlabeled and ^18^O-labeled peptides were based on peak heights in the MS1 spectra (Figure S1).

### 2.4. Antibody-Dependent Sperm Phagocytosis

U937 pro-monocytes were obtained from ATCC and cultured in an RPMI 1640 complete medium supplemented with L-glutamine, Fetal Bovine Serum (FBS), and Pen-Strep (GIBCO-11875093). Cells were seeded at a density of 0.5 × 10^6^/mL in 6-well plates containing sterile glass coverslips. Cells were treated with 100 ng/mL of phorbol-12-myristate-13-acetate (PMA) to stimulate macrophage differentiation for 48–72 h at 37 °C, after which activated U937 cells adhered to coverslips at a density of ~1 × 10^6^ cells per coverslip. Sperm cells were isolated from human semen samples of healthy men aged 18–45 years. All semen donors provided informed consent prior to collection (Human Subjects Protocols H36843).

The ADCP assay was performed as described by Oren-Benaroya et al. with modifications [34]. Briefly, 1 × 10^6^ sperm cells were suspended in sperm multipurpose handling media (MHM; FUJIFILM Irvine Scientific, Santa Ana, CA, USA) supplemented with either HCA-N, HCA_mRNA_, Campath (anti-CD52 IgG, positive control, Thermo Fisher Scientific Cat# MA5-16999, Waltham, MA, USA, RRID: AB_2538471), VRC01 (anti-HIV IgG, isotype control, produced in *Nicotiana* by ZabBio, Inc., San Diego, CA, USA), or medium only (a negative control). Sperm–antibody suspensions were added to wells and were incubated at 37 °C for 30 min. Following a wash step, cells were then incubated in PBS at 37 °C for 30 min and treated with trypsin for 5 min to remove non-specifically-bound sperm. The coverslips were retrieved and treated with a Differential Quik III dye kit (Polysciences, Warrington, PA, USA) to visualize phagocytosis under a light microscope. The number of associated antibody-opsonized sperm per macrophage was counted for each condition. Associated sperm were considered those either opsonized/engaged with the Fc receptor on the activated U937 cells (attached) or partially or completely engulfed.

### 2.5. Statistical Analysis

Data analysis and graph creation were performed using GraphPad Prism (Version 9.4.1; GraphPad Software Inc.; San Diego, CA, USA). Statistical significance between HCA-N and HCA_mRNA_ ADCP assay results was determined by a two-tailed paired *t*-test. Data were log-transformed prior to analysis. The definition of statistical significance was *p* < 0.05.

## 3. Results

### 3.1. Sequence Coverage Was Sufficient for Both HCA-N and HCA_mRNA_

We applied reversed-phase nanoUPLC-MS/MS to investigate the glycosylation of both HCA-N and HCA_mRNA_. Data obtained for HCA-N provided the high sequence coverage of HCA heavy and light chains with little to no contamination by other proteins (Table 1), owing to the fact that *Nicotiana* HCA production was performed in a commercial, GMP-grade facility (KBio, Inc., Owensboro, KY, USA). HCA_mRNA_, produced from cell culture in an academic lab setting, contained a high percentage of additional proteins. The high abundance of serotransferrin, which made up 66% of the sample, could be attributed to its endogenous expression by VK2 cells and subsequent transfer into the culture supernatant (Table S1). Serotransferrin is also among the 153 proteins produced in the FRT that are included in the “Normal Pap Test Core Proteome” [35]. Even so, the sequence coverage of HCA heavy and light chains in HCA_mRNA_ was sufficient to confirm their production, and the abundances of peaks corresponding to glycopeptides containing Asn^297^ were sufficient to generate a glycoform profile.

### 3.2. Compositional Differences in N-Glycan Profiles Were Observed between HCA-N and HCA_mRNA_

**Figure 3:**
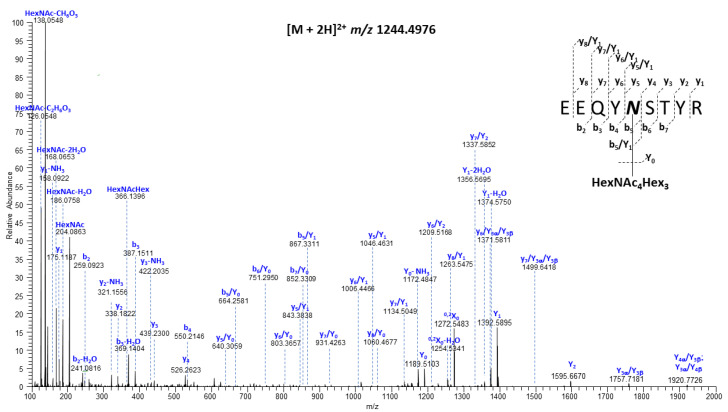
Representative HCD tandem mass spectrum of the [M + 2H]^2+^ precursor ion observed at *m*/*z* 1244.4976, corresponding to the *N*-glycopeptiform obtained from IgG HCA, consisting of the peptide ^293^EEQYNSTYR^301^, with its backbone modified at N^297^ by HexNAc_4_Hex_3_. The blue letters are designations of the fragment ion types, as introduced by Domon & Costello [36] and now the system universally used for this purpose.

Most human IgGs usually contain *N*-glycans only at the highly conserved *N*-glycosylation site on the Fc region of the heavy chain, Asn^297^. There are two potential *N*-glycosylation sites (NXS/T, X ≠ P) on the heavy chain of HCA (N^71^ and N^297^), but there are no predicted *N*-glycosylation sites on the light chain. We determined that the first heavy chain site is largely unoccupied (Figure S2). The second highly conserved glycan site is Asn^297^ (Figure 3). The presence of b- and y-type fragment ions provided the full peptide sequence information; the existence of the nearly complete series of b- and y-type product ions in this MS/MS spectrum allowed the assignment of the peptide sequence as ^293^EEQYNSTYR^301^. Moreover, the b_5_/Y_1_, y_5_/Y_1_ and y_5_/Y_2_ fragment ions (the b_5_ + HexNAc, y_5_ + HexNAc, y_5_ + HexAc_2_ ions, respectively) precisely defined the glycosylation site as N^297^.

**Figure 4:**
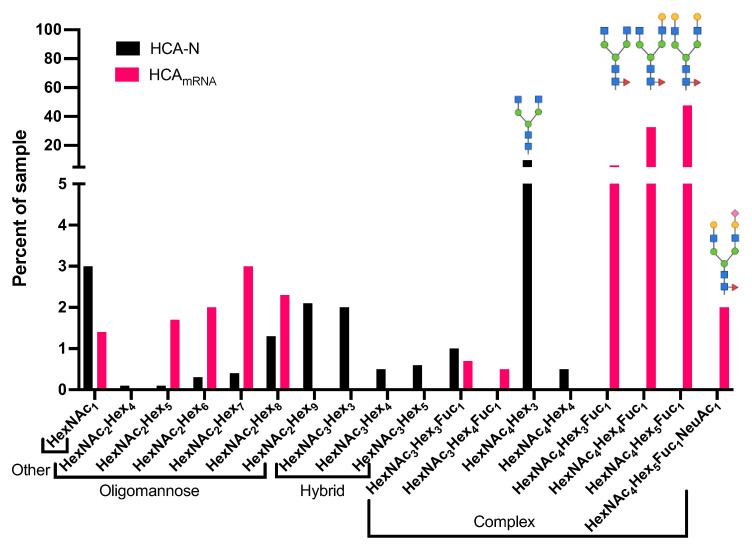
Composition of Fc *N*-glycans on platform-specific HCAs. Fc *N*-glycan profiles of HCA-N (black) and HCA_mRNA_ (pink). Glycans are located on the second potential *N*-glycosylation site on the HCA heavy chain (N^297^) with the amino acid sequence ^293^EEQY**N**STR^300^. The cartoons above selected bars each illustrate only one of the possible glycoforms corresponding to the compositions on the x-axis. Colored symbols representing monosaccharide units are defined on the right side of Figure 1.

Mass spectrometry-based glycoproteomic analysis revealed thirteen unique glycoforms of all three glycan types (i.e., oligomannose, hybrid, complex) at the Asn^297^ site on HCA-N (Figure 4). The quantitative analysis of *N*-glycosylation site occupancy revealed that only 49.1% of the ^293^EEQYNSTYR^301^ peptides was the N^297^ glycosylation site occupied by glycans (Table 2 and Figure S1). Among the peptides with an occupied site, all three glycan classes were well represented. Interestingly, despite the knockout of α1,3 FucT and the absence of other fucosyltransferases within the transgenic *Nicotiana* platform, around 1% of the sample was fucosylated, suggesting incomplete knockout. The most abundant glycan within this sample was G0, which is consistent with previous literature on *Nicotiana* manufacturing [33,37].

The Fc *N*-glycan profile of HCA-N was compared to HCA_mRNA_, which contained 11 glycoforms at the Asn^297^ site (Figure 4). Interestingly, the occupancy of this *N*-glycosylation site in HCA_mRNA_ was 96.0% (Table 2). All three glycan types were present; however, there was a bias toward complex types containing a fucose moiety. Specifically, the glycans of highest abundance were G0F, G1F, and G2F, which are all common among human IgGs. As mentioned earlier, core fucose can potentially reduce binding affinity to FcγRs [38]. Of note, HCA_mRNA_ contained a sialylated glycan (G2S1F), which is also known to reduce Fc receptor binding but promote a longer half-life. Thus, there were considerable differences between these platform-specific HCAs, not only in site occupancy but also in glycan types and glycoform distribution.

Additionally, we observed that monoHexNAc modified the Asn^297^ site of the Asn-Ser-Thr sequon of both HCA-N and HCA_mRNA_. This modification is denoted as “other” in Figure 4. Unlike a typical *N*-glycosylation of the Asn on NXS/T with a HexNAc_2_Hex_3_ core, the monoHexNAc as an *N*-glycan modifying the Asn^297^ site of this sequon is uncommon but probably results from lysosomal degradation.

### 3.3. HCA-N and HCA_mRNA_ Exhibit Different Levels of Sperm Phagocytosis Activation

**Figure 5:**
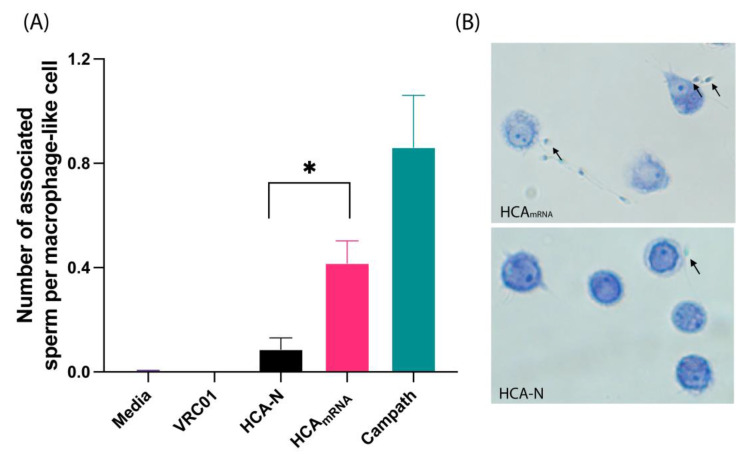
Antibody-dependent sperm phagocytosis of platform-specific HCAs. (**A**) Number of associated sperm per macrophage-like cell (i.e., engaged with Fcγ receptor/opsonized or partially internalized) for each antibody treatment (3.33 µg/mL). Assay controls are as follows: media-only negative control (*n* = 2), Campath-positive control (*n* = 2), and VRC01 isotype control (*n* = 1). HCA-N and HCA_mRNA_ data are expressed as the mean ± SEM of three independently performed experiments. HCA-N and HCA_mRNA_ log-transformed data were analyzed using a two-tailed paired *t*-test (* = *p* < 0.05, *p* = 0.0108). (**B**) Images of sperm cells associated with macrophage-like cells during the phagocytosis process. Images were taken at 200× magnification. Arrows point to sperm that are in the process of phagocytosis.

Both HCA-N and HCA_mRNA_ were capable of mediating sperm opsonization and phagocytosis at a low concentration of 3.33 µg/mL, though neither showed as robust activity as Campath, which is an anti-CD52 positive control for ADCP (Figure 5A,B). HCA_mRNA_ induced significantly more sperm phagocytosis than HCA-N (0.42 and 0.09 sperm per macrophage, respectively, *p* = 0.0108). We previously showed that at higher concentrations (e.g., 25–50 µg/mL), HCA-N can induce a greater degree of ADCP than seen here [16], but given that a large percentage of the Fc *N*-glycan site on HCA-N is unoccupied, as seen in Table 2, it is possible that, at a low concentration, there are not enough *N*-glycans present to interact with FcγRs and induce a stronger response, whereas HCA_mRNA_ is highly glycosylated and active. Overall, we demonstrated a biological difference, specifically a difference in Fc function, between the platform-specific HCAs.

## 4. Discussion

As the IgG-Fc glycan at residue Asn^297^ is needed for effector function through FcγR binding, it is important to characterize occupancy and glycoform distribution and to determine how they might differ among expression platforms. Here, we showed that HCAs expressed using two current production methods, transgenic *Nicotiana benthamiana* and mRNA-mediated expression in vaginal cells, have distinct Fc *N*-glycan profiles. The choice of mRNA expression in vaginal cells provides the most physiologically relevant in vitro model for studying the development of synthetic mRNA for contraception. Topical delivery of mRNA to the FRT would entail vaginal cellular uptake and subsequent mRNA translation. Mass spectrometry analysis confirmed that the Asn^71^ site on the heavy chain of HCA IgG is rarely occupied by glycan, while the Asn^297^ site carries a variety of glycoforms. In HCA-N, the most abundant glycoform at site N^297^ was G0. In HCA_mRNA_, most glycoforms were classified as the fucosylated complex type. Additionally, we observed that a glycoform bearing monoHexNAc was present at the Asn^297^ site of both HCA-N and HCA_mRNA_. The uncommon monoHexNAc modification is likely a product of lysosomal degradation; whether or not it has a biological function is unknown.

Each expression platform of HCA has its distinct advantages. On the one hand, mRNA-mediated expression of HCA in vaginal cells eliminates the need for the knockout of certain transferases for unwanted non-human glycans and confers native glycans to the antibody. The glycans on HCA_mRNA_ are produced by the host and, therefore, should be compatible with the host. On the other hand, we demonstrated that HCA-N had a simpler glycan profile than that of HCA_mRNA_; despite HCA-N containing more N^297^ glycoforms than HCA_mRNA_ (13 and 11, respectively), the distribution of these glycoforms was much tighter, favoring one glycoform in particular, compared to fairly evenly distributed glycoforms in HCA_mRNA_. A narrow glycoform distribution is a preferable characteristic because it facilitates functional consistency in antibodies.

We also found that HCA-N and HCA_mRNA_ facilitated varying levels of ADCP. This was not surprising since the *N*-glycan composition and biodistribution were distinct for each platform. Moreover, site occupancy data revealed that the majority of HCA_mRNA_ at site N^297^ was occupied by glycan (96%), compared to that of HCA-N (49.1%). This may have contributed to higher levels of ADCP that were observed using HCA_mRNA_ at a low concentration; however, it is unclear whether improved sperm phagocytosis is due to differences in glycoform distribution or glycan occupancy. Future studies are needed to investigate the functions of different glycoforms.

A recently completed phase 1 clinical trial of ZB-06, a vaginal film containing HCA, suggested that certain HCA Fc functions are important for contraception [23]. Notably, HCA mediates CDC [16], and high numbers of immotile sperm in cervical mucus in women using the ZB-06 film suggest that CDC may be an important contraceptive effect. HCA also mediates ADCP, which may be an undesirable Fc mechanism of HCA because antigen presentation of sperm proteins could lead to the host production of antisperm antibodies and contraceptive irreversibility. For this reason, the *Nicotiana* platform may be preferable since it exhibits lower levels of ADCP. The potential for ADCP to occur in vivo is still unclear and will be further evaluated in future HCA clinical trials, though we believe the probability is low due to reduced numbers of macrophages in the lower FRT compared to other tissues [39]. We provide evidence that different expression platforms, even for the same biologic, can impact both the bioactivity and safety profile. Therefore, when selecting an expression system for antibodies, platform-specific glycosylation patterns may be an important consideration among other parameters of characterization.

Glycan profiles of the Fc region are critical antibody quality attributes. For this reason, the FDA and other regulatory agencies require pharmaceutical companies to characterize and maintain protein drug glycosylation within defined acceptance criteria that limit their deviation from the pattern present in the drug candidate used in clinical trials [40]. Current efforts in glycoengineering and tailoring glycosylation patterns may facilitate the development of mAbs with more homogeneous IgG glycans that elicit the desired set of effector functions for their respective therapeutic uses [41]. Several methods are in development to tailor glycosylation. One method is through the enzymatic remodeling of IgG glycosylation, either by residue alteration via knockout or the recombinant expression of glycosyltransferases to achieve the addition or removal of the core fucose and/or sialic acids or through the use of transglycosylation via endoglycosidases and glycosynthases [42]. Another method is treatment with endoglycosidases, EndoS or EndoS2, for the hydrolysis and removal of the Fc glycan to block certain effector functions [43]. Though certain challenges and limitations still persist in this growing field, glycoengineering may be an important tool and future direction that could allow the optimization and homogenization of the Fc *N*-glycans on HCA produced within a given expression platform.

## Figures and Tables

**Table 1:** Sequence coverage and protein composition of HCA-N and HCA_mRNA_.

	Protein	Sequence Coverage	Percentage of Total Sample (%)
HCA-N	IgG heavy chain	99.60	72
	IgG Lambda light chain	80.80	24
HCA_mRNA_	IgG heavy chain	93.84	10
	IgG Lambda light chain	65.54	5
	Serotransferrin	80.69	66
	Bovine Serum Albumin	84.02	4
	Glutathione S transferase	84.69	2
	Keratin, Type II Cytoskeletal	45.10	2
	Clostridial Collagenase	40.10	<1
	Bovine Glutamate Dehydrogenase 1, Mitochondrial	47.00	<1

**Table 2:** Quantification of the Asn^297^ *N*-glycosylation site occupancy.

	HCA-N	HCA_mRNA_
Occupied	49.1%	96.0%
Not occupied	50.9%	4.0%

For the quantification of site occupancy, digested samples were dissolved in water (^18^O, 97%) containing 50 mM of NH_4_HCO_3_ and deglycosylated with PNGase F prior to MS analysis.

## Data Availability

The nUPLC-MS/MS data presented in this study are openly available in ProteomeXchange with reference number PXD047910.

## Supplementary Materials

The following supporting information can be downloaded at: https://www.mdpi.com/article/10.3390/antib13010017/s1, Table S1: Protein composition of untransfected VK2 culture supernatant; Figure S1: Mass spectrometric relative quantification of unmodified, deamidated, and deglycosylated peptides that contain the IgG Asn^297^ potential *N*-glycosylation site, after PNGase glycan release in the presence of H_2_^18^O, determined for (A) HCA-N and (B) HCA_mRNA_; Figure S2: HCD tandem mass spectrum of the [M + 3H]^3+^ precursor ion at *m*/*z* 782.0487, ^63^GLEWIGEINHSGSTNYNPSLR^83^, obtained from IgG HCA, corresponding to the tryptic peptide containing the unoccupied potential *N*-linked site at N^71^ (NHS).
