# Supplementary material for: Platform-Specific Fc N-Glycan Profiles of an Antisperm Antibody
Source: Antibodies (Basel). 2024 Mar 6;13(1):17. doi: 10.3390/antib13010017 (PMC10967333; doi:10.3390/antib13010017)
Supplement: Supplementary file 1 [file antibodies-13-00017-s001.zip › antibodies-2846600-supplementary.pdf]

Supporting Information

Platform-Specific Fc *N*-Glycan Profiles of an Antisperm Antibody

Ellena Nador [1], Chaoshuang Xia [2], Philip J. Santangelo [3], Kevin J. Whaley [4], Catherine E. Costello [2,*] and Deborah J. Anderson [1,*]

[1] Department of Medicine, Boston University Chobanian & Avedisian School of Medicine, Boston, MA 02118, USA
[2] Center for Biomedical Mass Spectrometry, Boston University Chobanian & Avedisian School of Medicine, Boston, MA 02118, USA
[3] Wallace H. Coulter Department of Biomedical Engineering, Emory University, Atlanta, GA 30322, USA
[4] ZabBio, Inc., San Diego, CA 92121, USA
[*] Correspondence: cecmsms@bu.edu (C.E.C.); deborah.anderson@bmc.org (D.J.A.)

Table S1. Protein composition of untransfected VK2 culture supernatant.

Protein	Percentage of total sample	Sequence coverage (%)	Molecular weight of unmodified protein (kDa)
Serotransferrin	97	66	77
HCA IgG Lambda Heavy Chain	<1	11.2	48
HCA IgG Lambda Light Chain	<1	9.9	26
Bovine Serum Albumin	1	78.9	66
Trypsin	1	20.8	24

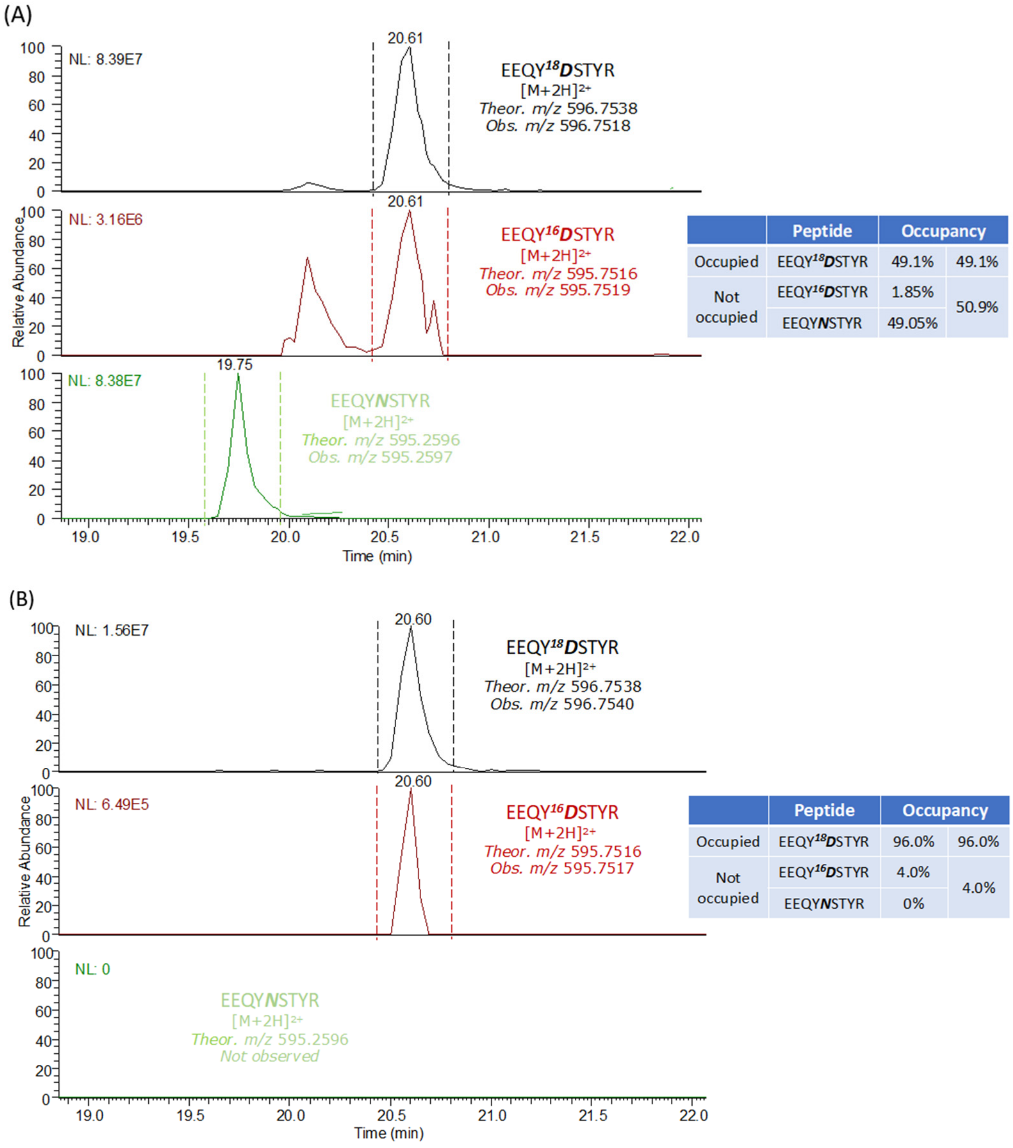

(A)

	Peptide	Occupancy	
Occupied	EEQY^{18}**D**STYR	49.1%	49.1%
Not occupied	EEQY^{16}**D**STYR	1.85%	50.9%
	EEQY**N**STYR	49.05%	

(B)

	Peptide	Occupancy	
Occupied	EEQY^{18}**D**STYR	96.0%	96.0%
Not occupied	EEQY^{16}**D**STYR	4.0%	4.0%
	EEQY**N**STYR	0%	

Figure S1. Mass spectrometric relative quantification of unmodified, deamidated, and deglycosylated peptides that contain the IgG Asn^{297} potential *N*-glycosylation site, after PNGase glycan release in the presence of $H_2{}^{18}O$, determined for (A) HCA-N and (B) HCA$_{mRNA}$.

For each, the extracted ion chromatograms (EICs) of the unmodified asparagine (N) (lowest panel), the aspartic acid generated by deamidation (^{16}D) (middle panel), and aspartic acid generated by PNGase-mediated glycosylation in the presence of $H_2{}^{18}O$ (^{18}D) (upper panel) are shown. Only the occupied sites gain an ^{18}O label. All peptide assignments were confirmed based on charge state and peptide fragment ions in the corresponding MS2 spectrum.

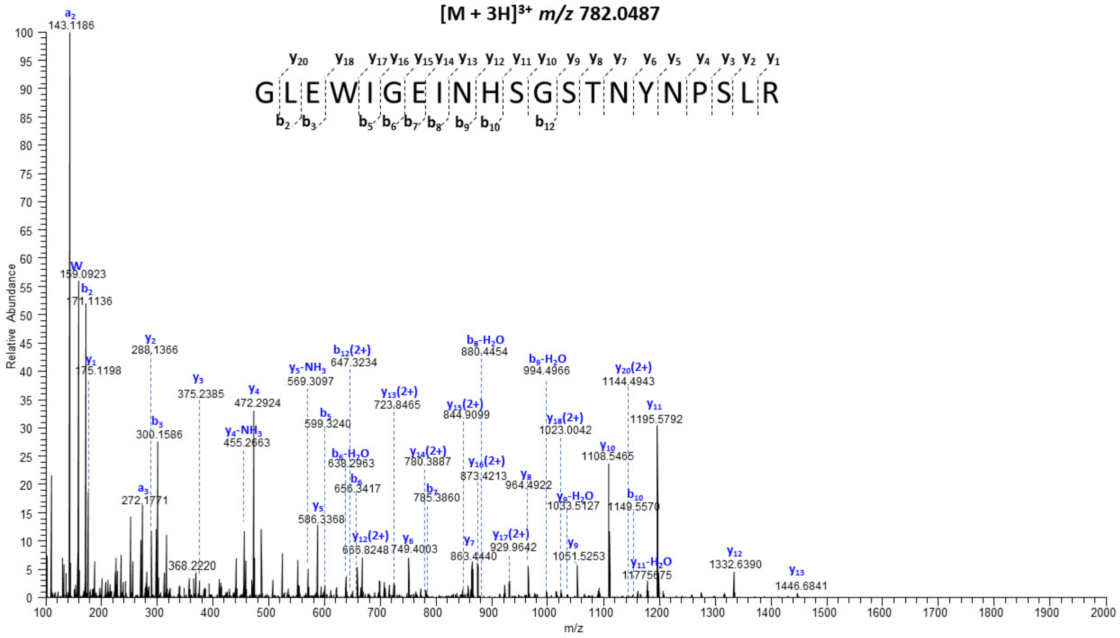

Figure S2. HCD tandem mass spectrum of the [M + 3H]$^{3+}$ precursor ion at *m/z* 782.0487, ^{63}GLEWIGEINHSGSTNYNPSLR^{83}, obtained from IgG HCA, corresponding to the tryptic peptide containing the unoccupied potential *N*-linked site at N^{71} (NHS).

N^{75} is not part of an NXS/T sequon and N^{77} is not a potential glycosylation site, since Pro follows Asn. The blue letters are designations of the fragment ion types, as introduced by Domon & Costello [36] and now the system universally used for this purpose.
